# Dose dependent toxicity of streptozotocin inducing diabetic complications in male Sprague Dawley rats with metabolic biochemical histopathological effects

**DOI:** 10.1038/s41598-025-27100-y

**Published:** 2025-12-08

**Authors:** Ahmed A. Sedik, Aliaa E. M. K. El-Mosallamy, Noha N. Yassen, Nesma M. E. Abo El-Nasr

**Affiliations:** 1 Pharmacology Department, Medical Research and Clinical Studies Institute, National Research Centre, El-Buhouth St., Dokki, Cairo 12622 Egypt; 2 Pathology Department, Medical Research and Clinical Studies Institute, National Research Centre, El-Buhouth St., Dokki, Cairo 12622 Egypt

**Keywords:** Diabetes mellitus, Dose regimen, Streptozotocin, Diabetic complications, Rats, Biochemistry, Cardiology

## Abstract

Diabetes mellitus (DM) is a chronic metabolic disorder characterized by persistent hyperglycemia, leading to microvascular and macrovascular complications including cardiomyopathy, retinopathy, neuropathy, and nephropathy. This study evaluated the diabetogenic potential and pathological outcomes of two single intraperitoneal doses of streptozotocin (STZ) in male Sprague Dawley rats. Twenty-four rats (200–220 g) were randomly assigned to three groups (n = 8/group): control, STZ 45 mg/kg, and STZ 60 mg/kg. DM was confirmed 72 h post-injection via elevated serum glucose. At the end of the experiment, blood and tissue samples were collected to assess metabolic parameters, lipid profile, liver function, oxidative stress markers, inflammatory cytokines, and histopathological changes. Both doses induced significant hyperglycemia, elevated glycated hemoglobin, and reduced insulin levels. Diabetic rats also showed dyslipidemia, liver dysfunction, increased oxidative stress (↓GSH, ↑MDA), and elevated inflammatory markers (TNF-α, il-6). Histopathological and morphometric analyses confirmed tissue damage and biochemical alterations. Notably, the 60 mg/kg dose produced a more severe and consistent diabetic phenotype, with greater metabolic derangements, oxidative injury, inflammation, and organ pathology compared to the 45 mg/kg dose. These findings suggest that while both doses effectively induce experimental diabetes, 60 mg/kg STZ provides a more robust model for studying DM-associated complications in vital organs.

## Introduction

Diabetes Mellitus (DM) is a chronic global metabolic disorder characterized by persistently elevated blood sugar levels as well as disturbances in fat, carbohydrates and protein metabolism subsequently ensue the insulin resistance due to defects in insulin synthesis or secretion and insulin action, or both^1^. Diabetes mellitus is categorized into two major etiologies: insulin deficiency (Type-1 DM, mature-onset diabetes in the young and insulin resistance (Type-2 DM, gestational DM, etc.). The prevalence of diabetes has been steadily increasing in recent decades, according to the International Diabetes Federation (IDF) The current number of diagnosed cases is ~over 422 million Evidence people global had diabetes in 2014, expected to rise to ~592 million by 2035^2^.

Diabetes economically drains the global healthcare systems^3^. The International Diabetes Federation (IDF) revealed that 537 million people worldwide had diabetes in 2021, globally causing health expenditures of US$966 billion, forecast to reach more than $1054 billion by 2045^4^. It significantly impacts both morbidity and mortality, posing substantial health risks globally. Where, it is associated with serious complications such as cardiovascular disease, chronic kidney disease, neuropathy, and retinopathy due to the prolonged exposure of many vital organs to elevated blood glucose levels and formation of free radicals which are concerned in the pathogenesis of the diseases and development of its complications^5^. These complications contribute to increased morbidity by causing chronic pain, loss of function, and reduced life quality^6^. Moreover, diabetes mellitus is a major contributor to premature mortality, largely due to its association with cardiovascular diseases, kidney failure, and an increased risk of certain cancers^7,8^. Consequently, effective management and preventive strategies are crucial in reducing the disease’s burden on individuals and healthcare systems.

Streptozotocin (STZ) is a widely used agent for inducing experimental diabetes mellitus (DM) in rodents due to its selective cytotoxicity to pancreatic β-cells^9^. Numerous studies have established single-dose intraperitoneal administration of STZ as a reliable model for Type 1 diabetes, with commonly used doses ranging between 40 and 70 mg/kg. For instance, Szkudelski^10^ reviewed the diabetogenic mechanisms of STZ and emphasized its dose-dependent action but did not compare specific dosing regimens in a controlled setting. Similarly, Rakieten et al.^11^, among the earliest to demonstrate STZ’s β-cell toxicity, used high-dose injections (above 65 mg/kg), which, while effective, are now considered to have high mortality risks and systemic toxicity. More recent studies, such as Furman^13^, have employed that STZ (60 mg/kg) to reliably induce hyperglycemia but often overlook long-term variability in glycemic control or lack comparative data with lower doses from STZ like (45 mg/kg)^12^. On the other hand, investigations by Lenzen^12^ and Al-Awar et al.^14^ explored a broader range of doses but did not standardize important parameters such as fasting duration, timing of glucose measurement, or post-induction follow-up, which reduces comparability^13,14^.

Despite the frequent use of both 45 mg/kg and 60 mg/kg STZ doses in the literature, few studies have directly compared their diabetogenic potential under consistent experimental conditions. This presents a critical gap, as the choice of dose has significant implications for the severity and stability of the diabetic state, animal survival, and subsequent pathophysiological outcomes. The present study addresses these limitations by directly evaluating the efficacy and safety of a single intraperitoneal injection of STZ at 45 mg/kg and 60 mg/kg in rats, under standardized conditions. This approach enables a more precise determination of the optimal dose for inducing Type 1-like diabetes with minimal variability and maximum reproducibility, thereby enhancing the translational value of the model for future therapeutic investigations.

## Material and methods

### Animals

Adult male Sprague-Dewly rats, weighing 200–220 gm were obtained from Animal breeding unit, National Research Centre, Dokki, Giza. Rats were housed in stainless steel cages at room temperature, good ventilation and received water and commissural rat chow diet. They were left for two weeks to acclimatize before starting the experiment. The study received approval from the Medical Research Ethics Committee (MREC) of the National Research Centre, with the assigned approval number (130601101) and all methods were performed in accordance with the relevant scientific guidelines and regulations. The current study is reported in accordance with ARRIVE guidelines.

### Chemicals

STZ was purchased in the form of vial contain 100 mg STZ powder from Sigma‒Aldrich, USA (CAS number: 605-19-2; 99.7% pure). STZ was freshly dissolved immediately prior to injection in ice‑cold 0.1 M sodium citrate buffer (pH 4.5) Rats were administered a single intraperitoneal injection of STZ at doses of 45 mg/kg or 60 mg/kg body weight. To prevent early hypoglycemia, all animals received access to a 5% glucose solution overnight following STZ administration.

### Experimental groups

Twenty- Four Adult male Sprague-Dawly rats were randomly separated into three groups (8 rats/group). Group I (Control group): Normal rats fed on commissural rat chow diet and received 0.5 ml distilled water for 28 days. Group II (STZ group-45 mg/kg): Rats of this group subjected to I.P. injection of single dose of STZ (45 mg /kg body weight) and were allowed to feed on commissural rat chow diet and all rats received 0.5 ml distilled water for 28 days^15^. Group III (STZ group- 60 mg/kg): Rats of this group subjected to I.P. injection of single dose of STZ (60 mg /kg body weight) and were allowed feed on commissural rat chow diet and all rats received 0.5 ml distilled water for 28 days^16^.

At the end of the study, and after anaesthesia with ketamine (100 mg/kg) and xylazine (10 mg/kg)^17^, blood samples were collected from the eyes of the retro-orbital plexus of the rats^18^. Sera were separated by centrifugation at 3000 rpm for 15 min at 4 °C using a refrigerated centrifuge (Laborezentrifuger 2k15, Sigma, Germany)^19^. The levels of serum biochemical parameters were measured using commercially available kits; Serum glucose was measured using a glucose colorimetric assay kit (Cat. No. ab65333, Abcam, Cambridge, UK). Serum insulin was determined using an ELISA kit (Cat. No. E-EL-R2466, Elabscience, Wuhan, China)^20^. Glycated hemoglobin (HbA1c) was assessed using a glycated hemoglobin assay kit (Cat. No. 80300, Crystal Chem, Elk Grove Village, IL, USA). AST and ALT activities were measured using commercial enzymatic assay kits (Cat. No. MAK055 and MAK052, respectively, Sigma-Aldrich, St. Louis, MO, USA).

Total cholesterol, triglycerides (TG), HDL, and LDL levels were quantified using a lipid profile kit (Cat. No. CH200, TG100, HDL200, and LDL200, respectively, BioAssay Systems, Hayward, CA, USA)^21^. Malondialdehyde (MDA) levels were assessed using a lipid peroxidation (MDA) assay kit (Cat. No. K739-100, BioVision, Milpitas, CA, USA)^22,23^.Tumor necrosis factor-alpha (TNF-α) and interleukin-6 (il-6) were determined using ELISA kits (TNF-α: Cat. No. SEA133Ra; il-6: Cat. No. SEA079Ra, Cloud-Clone Corp., Wuhan, China)^24^.

#### Histological studies

The rats were euthanized through cervical dislocation and their organs (liver, kidney, lung and brain) were dissected immediately. The specimens were then fixed in 10% neutral-buffered formalin for 72 h at least. All the specimens were washed in tap water for half an hour and then dehydrated in ascending grades of alcohol, cleared in xylene and embedded in paraffin^25^.

#### Staining for general morphology

Serial sections of 5 μm thick were cut and stained with haematoxylin and eosin for histopathological investigation^26^.

#### Special stains

Serial sections were cut and stained by Mallory trichrome for detection of the connective tissue in blue colour and Toluidine blue which stains nucleic acids dark blue^27,28^.

#### Histological examination and morphometric analysis

Hematoxlyin and eosin slides were examined and captured at the pathology lab, National research Centre using light microscope Olympus CX41 and SC100 video camera attached to a computer system. Photomicrographes were taken at different magnifications and processed using Adobe Photoshop version8.0. Further histopathological evaluation was done using quantitative morphometric analysis of the pathological changes. The amount of connective tissue and nucleic acids using histochemical stains Mallory trichrome and Toluidine blue, respectively assessed by detection of area % of these contents were determined using a computer-assisted automated image analyzer. A Leica Q Win image processing and analysis system (Cambridge, UK, Image Analyzer Unit; Pathology Department, National Research Centre, Cairo, Egypt) was used for interactive automatic measurement of the percentage of detected areas on the stained slides by analyzing ten random fields per slide. The expression of histochemical stains was evaluated in the entire section at a magnification of × 200.

## Statistical analysis

One-way analysis of variance (ANOVA) was used for all quantifiable comparisons in our study, and Tukey’s multiple comparison test was performed using the GraphPad Prism program 8.0, USA). Results are presented as mean ± SD (8 rats) and the difference was documented significant when *p* value is ≤ 0.05.

## Results

### Effect of different administration of STZ on the serum glucose, insulin and glycated hemoglobin levels in male Sprague-Dawley rats

**Fig. 1:**
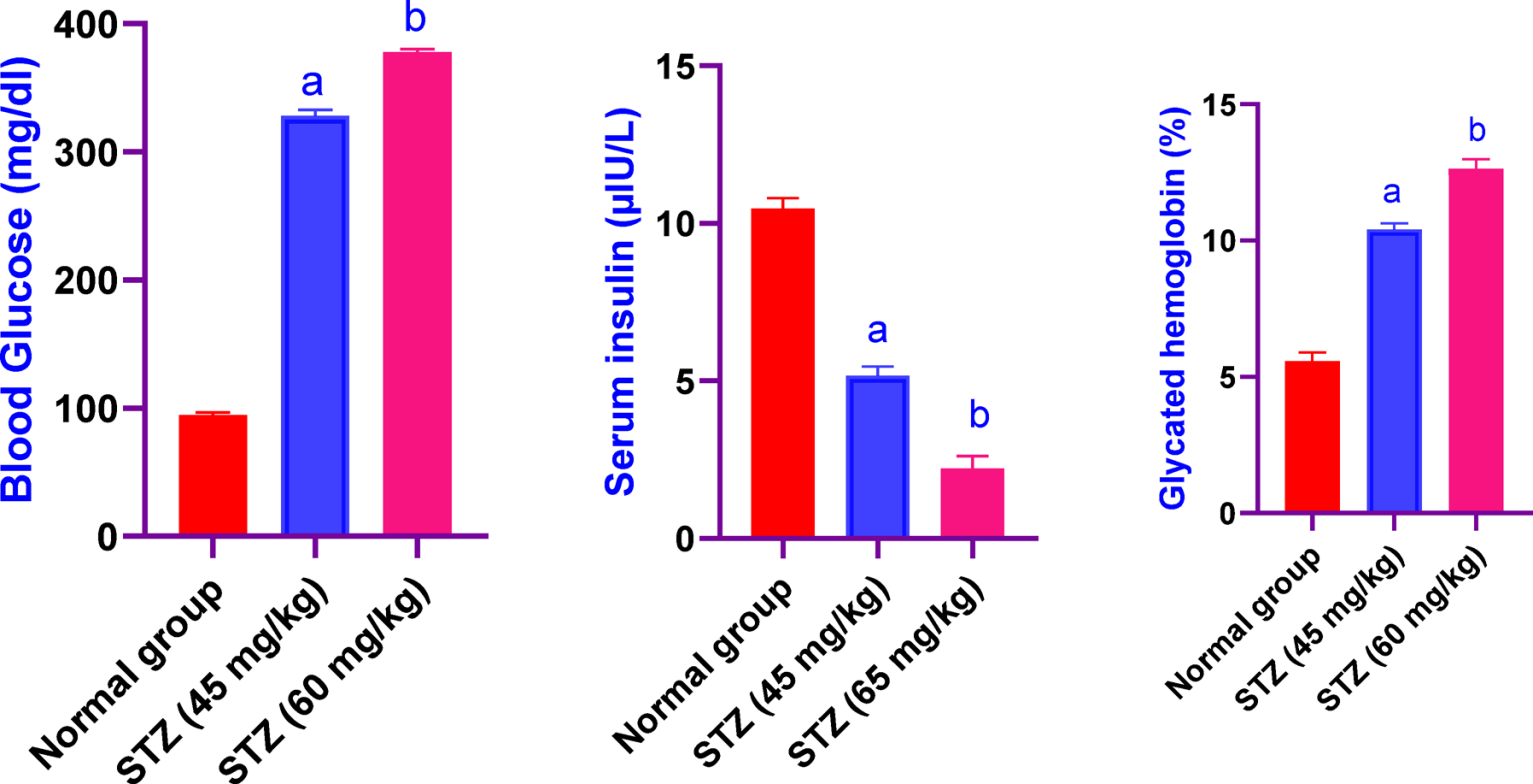
Effect of different administration of STZ on the Serum glucose, insulin and glycated hemoglobin levels in male Sprague-Dawley rats. Rats were received single dose of STZ (45 or 60 mg/kg i.p.) to induce DM. Serum glucose, insulin and glycated hemoglobin was evaluated. Results are expressed as mean ±SD (n = 8). ^a^Significant difference from normal group *p < 0.05*. ^b^Significant difference from STZ (45 mg/kg) group *p < 0.05*.

Rats received STZ (45 mg/kg) showed an increase in the levels of serum glucose and glycated hemoglobin levels nearly, 3.5 fold and 1.6 fold, respectively, also, a decrease in insulin values by, 49%, as compared to the control group rats. Also, rats in group (STZ 60 mg/kg) revealed a significant increase in serum glucose and glycated hemoglobin by, 3.9 fold and 2.2 fold, respectively, also, a significant reduction in the values of insulin by, 21%, as compared to the control group rats (Fig. 1).

### Effect of different administration of STZ on the serum values of triglycerides and cholesterol in male Sprague-Dawley rats

**Fig. 2:**
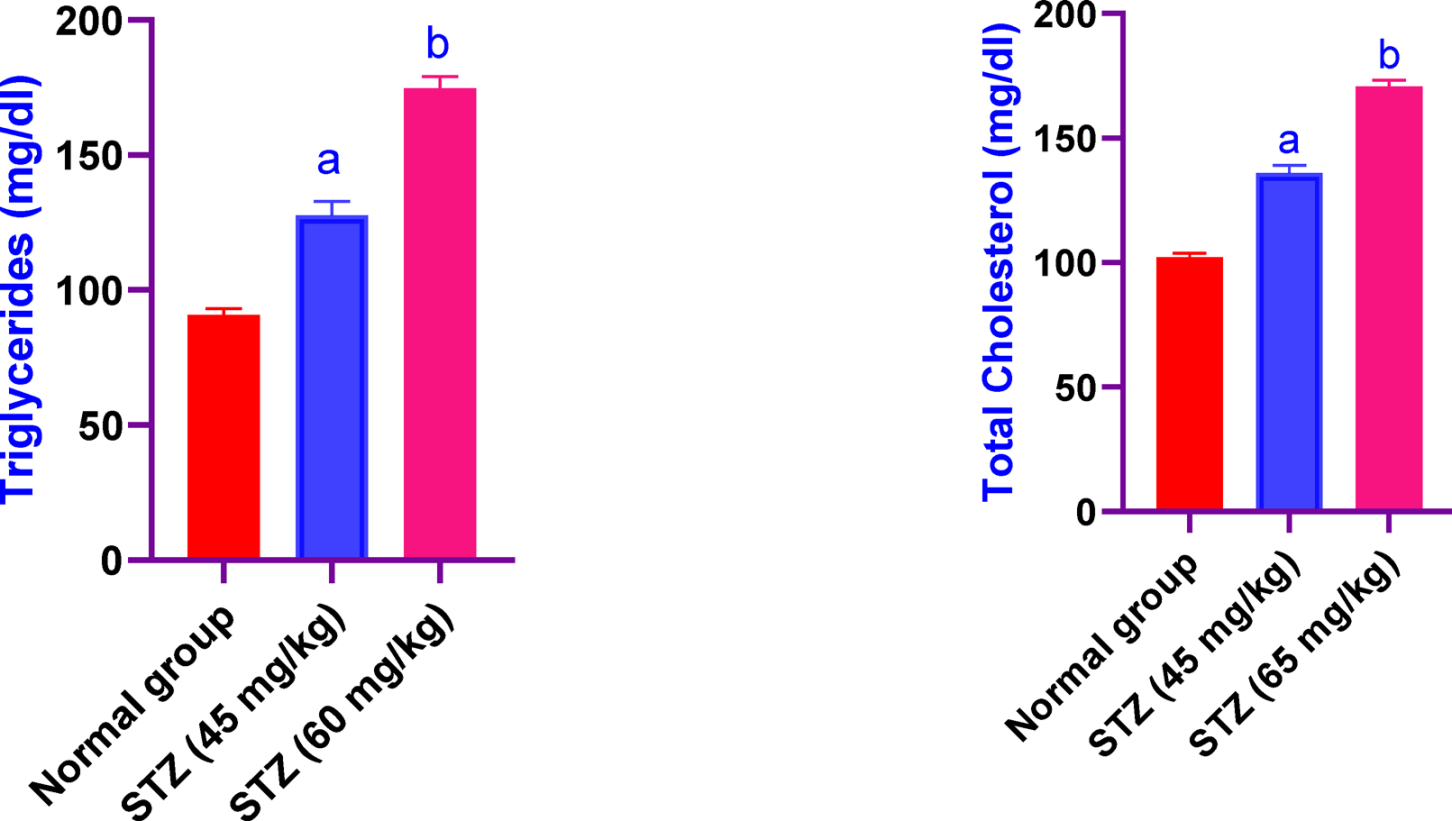
Effect of different administration of STZ on the serum values of triglycerides and cholesterol in male Sprague-Dawley rats. Rats were received single dose of STZ (45 or 60 mg/kg i.p.) to induce DM. Serum values of triglycerides and cholesterol was evaluated. Results are expressed as mean ± SD (n = 8). ^a^Significant difference from normal group *p < 0.05*. ^b^Significant difference from STZ (45 mg/kg) group *p < 0.05*.

Serum triglyceride and cholesterol levels were significantly elevated in both STZ-treated groups. Compared to control rats, triglycerides increased by approximately 1.4- and 1.9-fold in the STZ-45 and STZ-60 groups, respectively, while total cholesterol rose by 1.3- and 1.7-fold (Fig. 2). Absolute values (mean ± SD) are detailed in Table 1: triglycerides were 90.67 ± 4 mg/dl (control), 127.7 ± 9 mg/dl in STZ-45, and 174.7 ± 7.6 mg/dl in STZ-60. Similarly, total cholesterol levels were 102.1 ± 2.9 mg/dl (control), 135.8 ± 5.5 mg/dl in STZ-45, and 170.7 ± 4.5 mg/dl in STZ-60), confirming a clear dose-dependent trend.

**Table 1:** Effect of different administration of STZ on the serum values of triglycerides and cholesterol in male Sprague-Dawley rats.

	Triglycerides (mg/dl)	Total cholesterol (mg/dl)
Normal Group	90.67 ± 4	102.1 ± 2.9
STZ 45 mg/kg	127.7 ± 9^a^	135.8 ± 5.5^a^
STZ 60 mg/kg	174.7 ± 7.6^b^	170.7 ± 4.5^b^

Rats were received single dose of STZ (45 or 60 mg/kg i.p.) to induce DM. Serum values of triglycerides and cholesterol was evaluated. Results are expressed as mean ± SD (n = 8). ^a^Significant difference from normal group *p* < *0.05*. ^b^Significant difference from STZ (45mg/kg) group *p* < *0.05.*

### Effect of different administration of STZ on the serum values of HDL and LDL in male Sprague-Dawley rats

**Fig. 3:**
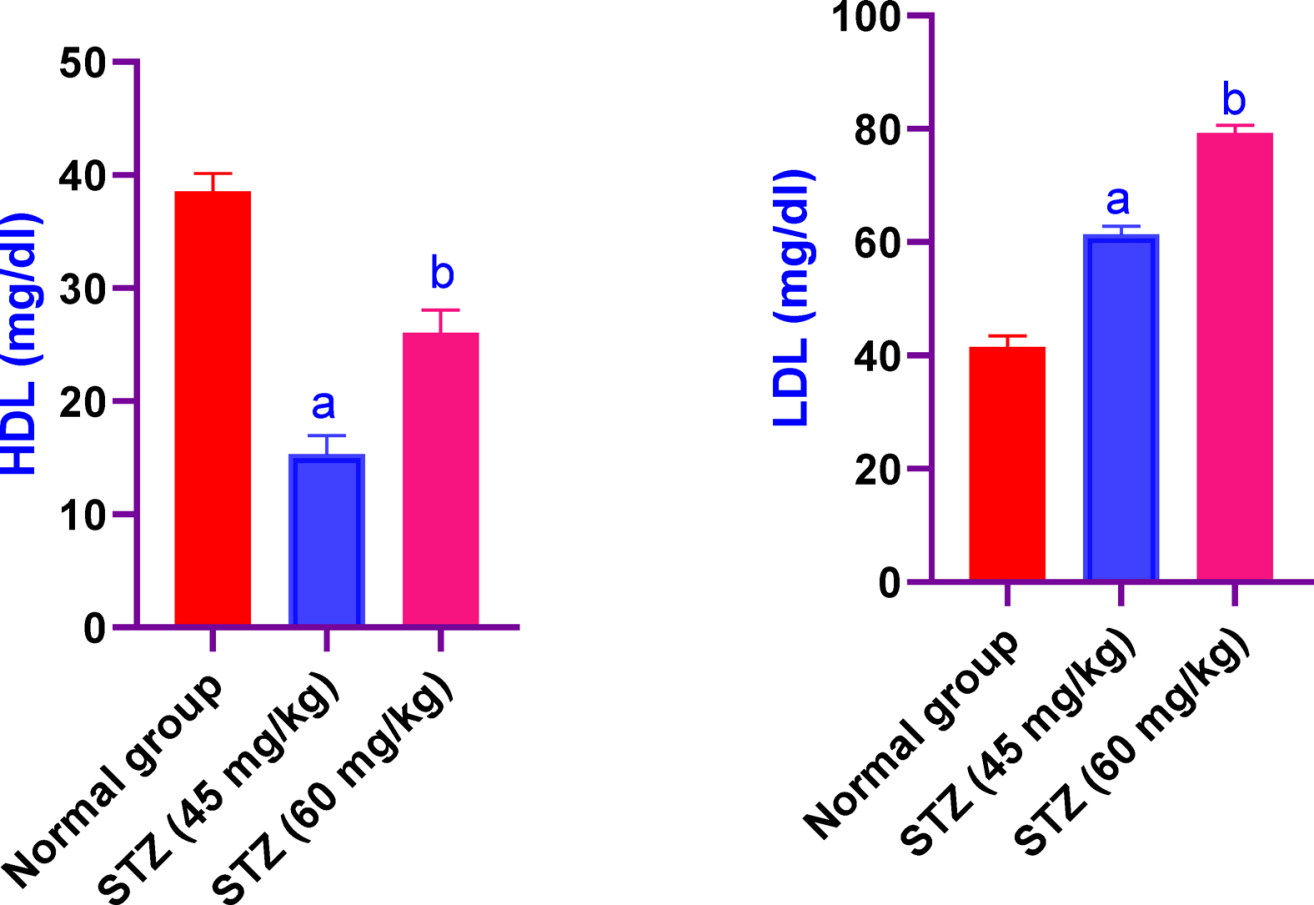
Effect of different administration of STZ on the serum values of HDL and LDL in male Sprague-Dawley rats. Rats were received single dose of STZ (45 or 60 mg/kg i.p.) to induce DM. Serum values of HDL and LDL was evaluated. Results are expressed as mean ± SD (n = 8). ^a^Significant difference from normal group *p < 0.05*. ^b^Significant difference from STZ (45 mg/kg) group *p < 0.05*.

Serum values of HDL in male Sprague-Dawley rats were significantly decreased in rats received STZ 45 and 60 mg/kg, by 38% and 66%, respectively, as compared to the control group rats. While, the values of LDL were significantly increased in rats received STZ 45 and 60 mg/kg, by 145% and 189%, respectively, as compared to the control group rats (Fig. 3).

### Effect of different administration of STZ on the serum values of AST and ALT in male Sprague-Dawley rats

**Fig. 4:**
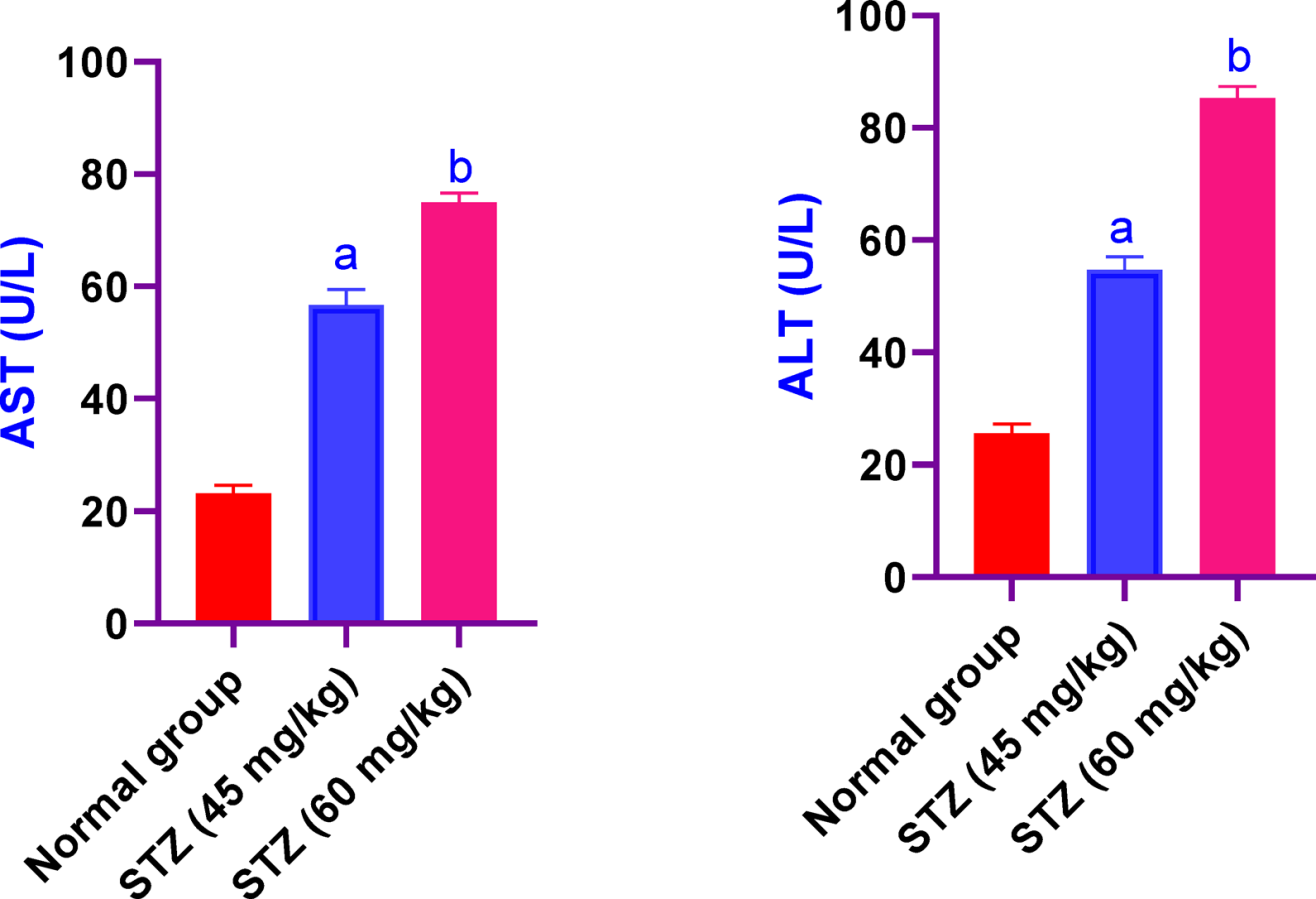
Effect of different administration of STZ on the serum values of AST and ALT in male Sprague-Dawley rats. Rats were received single dose of STZ (45 or 60 mg/kg i.p.) to induce DM. Serum values of AST and ALT was evaluated. Results are expressed as mean ± SD (n = 8). ^a^Significant difference from normal group *p < 0.05*. ^b^Significant difference from STZ (45 mg/kg) group *p < 0.05*.

In male Sprague-Dawley rats, treatment with STZ at both 45 and 60 mg/kg resulted in significant elevations of serum AST and ALT. Compared to controls, AST increased approximately 2.5- and 3.3-fold, while ALT rose by around 2.1- and 3.3-fold, respectively (Fig. 4). Absolute enzyme activities (mean ± SD) are shown in Table 2: control rats exhibited AST 23 ± 2 U/L and ALT 26 ± 3 U/L, STZ-45 rats showed AST 57 ± 5 U/L and ALT 55 ± 4 U/L, and STZ-60 rats had AST 75 ± 3 U/L and ALT 85 ± 4 U/L. Additionally, the STZ-60 group had significantly higher AST and ALT levels than the STZ-45 group (^b^*p* < 0.05), confirming a dose-dependent effect**.**

**Table 2:** Effect of different administration of STZ on the serum values of AST and ALT in male Sprague-Dawley rats.

	AST(U/L)	ALT(U/L)
Normal group	23 ± 2	26 ± 3
STZ 45 mg/kg	57 ± 5^a^	55 ± 4^a^
STZ 60 mg/kg	75 ± 3^b^	85 ± 4^b^

Rats were received single dose of STZ (45 or 60 mg/kg i.p.) to induce DM. Serum values of AST and ALT was evaluated. Results are expressed as mean ± SD (n = 8). ^a^Significant difference from normal group *p* < *0.05*. ^b^Significant difference from STZ (45mg/kg) group *p* < *0.05.*

### Effect of different administration of STZ on the serum values of GSH and MDA in male Sprague-Dawley rats

**Fig. 5:**
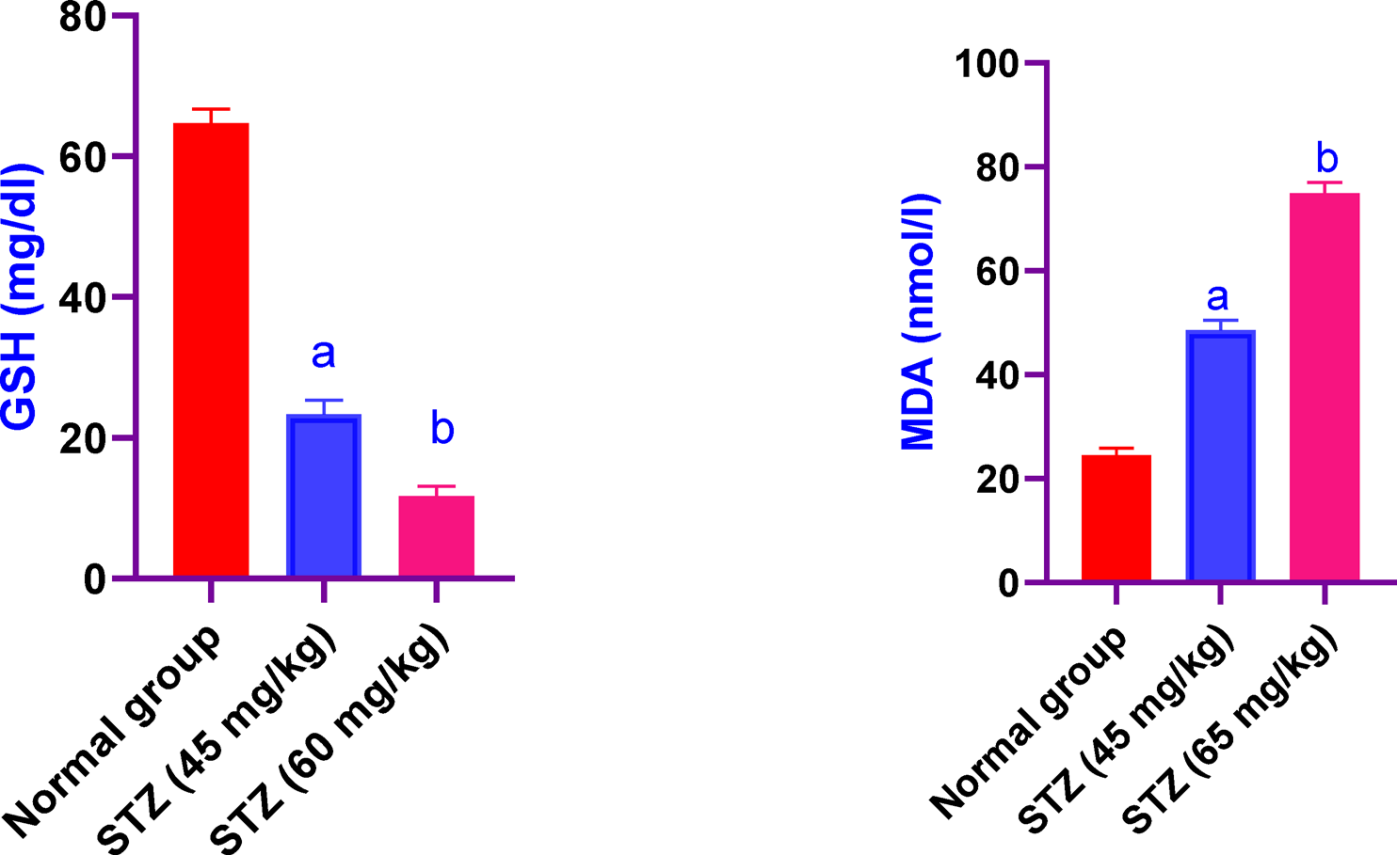
Effect of different administration of STZ on the serum values of GSH and MDA in male Sprague-Dawley rats. Rats were received single dose of STZ (45 or 60 mg/kg i.p.) to induce DM. Serum values of GSH and MDA was evaluated. Results are expressed as mean ± SD (n = 8). ^a^Significant difference from normal group *p < 0.05*. ^b^Significant difference from STZ (45 mg/kg) group *p<0.05*.

Serum values of GSH in male Sprague-Dawley rats were significantly decreased in rats received STZ 45 and 60 mg/kg, by 18% and 36%, respectively, as compared to the control group rats. While, the values of MDA were significantly increased in rats received STZ 45 and 60 mg/kg, by 1.9fold and threefold, respectively, as compared to the control group rats (Fig. 5).

### Effect of different administration of STZ on the serum values of TNF- α and il-6 in male Sprague-Dawley rats

**Fig. 6:**
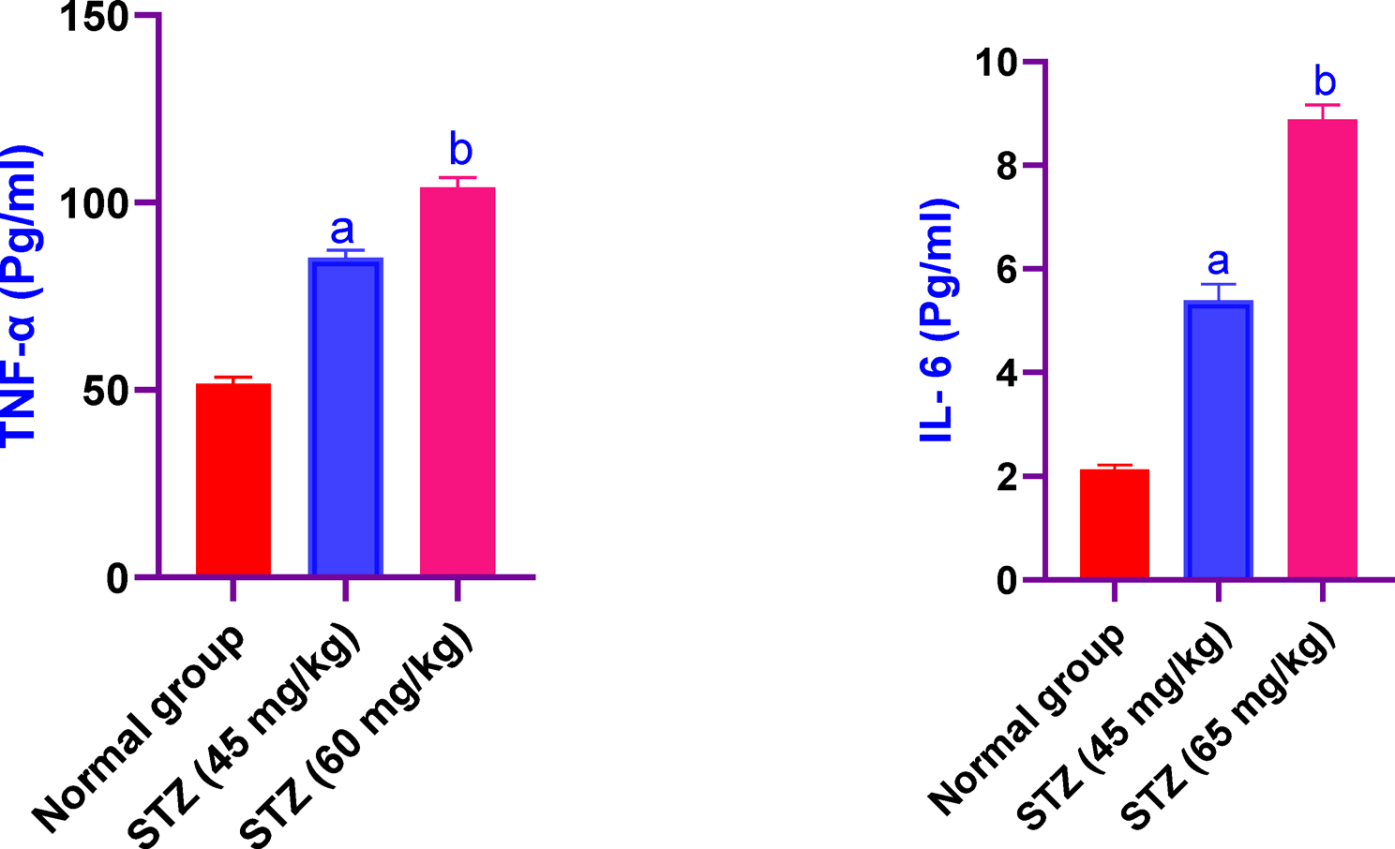
Effect of different administration of STZ on the serum values of TNF- α and il-6 in male Sprague-Dawley rats. Rats were received single dose of STZ (45 or 60 mg/kg i.p.) to induce DM. Serum values of TNF-α and IL-6 was evaluated. Results are expressed as mean ± SD (n = 8). ^a^Significant difference from normal group *p < 0.05*. ^b^Significant difference from STZ (45 mg/kg) group *p < 0.05*.

Regarding the serum values of TNF- α in male Sprague-Dawley rats were significantly increased in rats received STZ 45 and 60 mg/kg, by 1.6 fold and twofold. Also, the serum values of and il-6 in rats received STZ 45 and 60 mg/kg, by 2.5fold and fourfold, respectively, as compared to the control group rats (Fig. 6).

### Histopathological examination for the effect of different administration of STZ on male Sprague-Dawley rats

#### Histopathological picture of the liver tissue

**Fig. 7:**
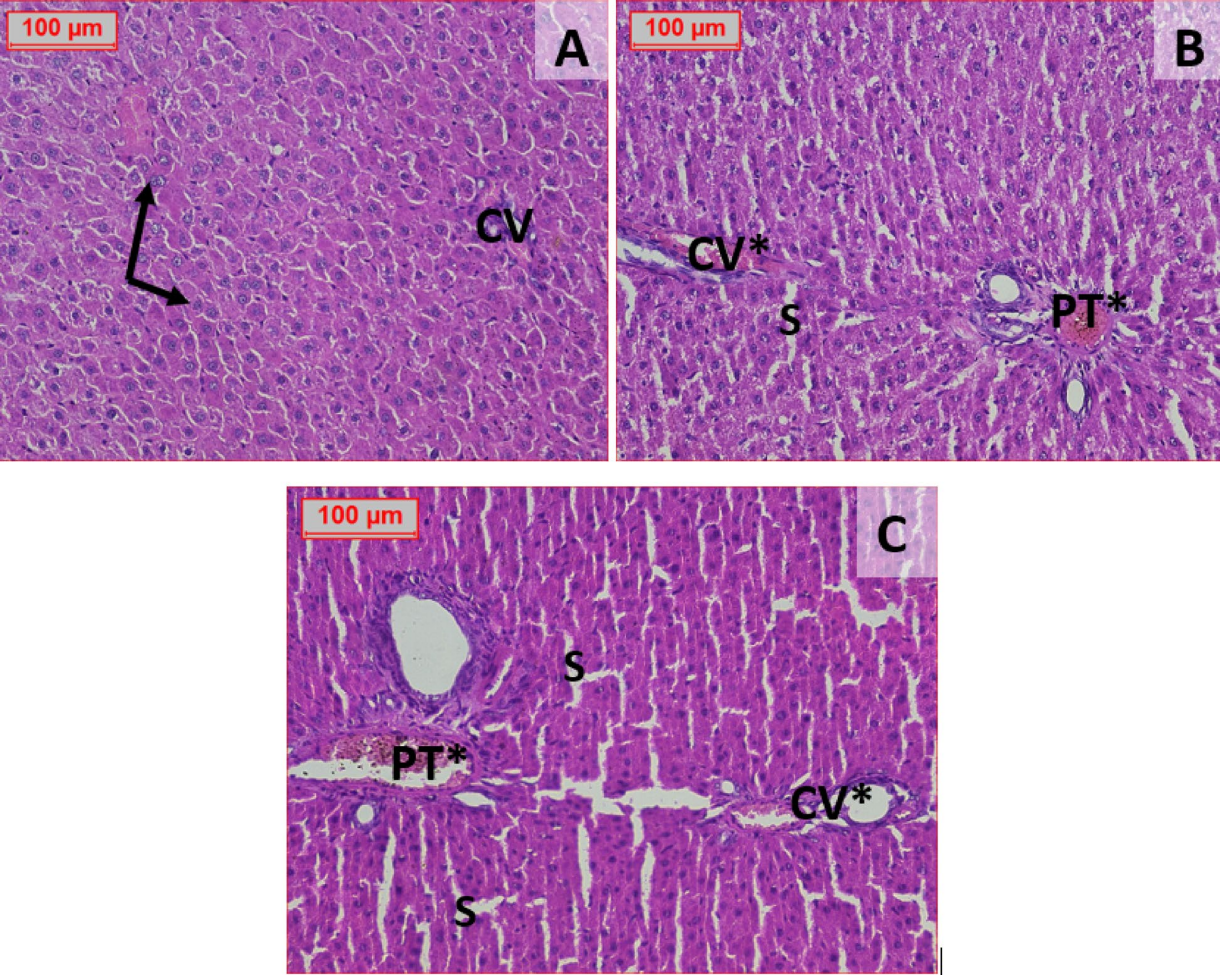
Effect of different administration of STZ on the histopathological picture of the liver of male Sprague-Dawley rats. Illustrated sections of the liver tissue for all tested groups. The normal control group shows a normal-appearing central vein and portal tract, formed of the portal vein, hepatic artery, and bile duct, with normal sinusoids and healthy hepatocytes having central vesicular nuclei; some hepatocytes show a prominent nucleolus (**A**). The drug-treated groups (two different doses) show dilated congested blood vessels; dilated congested central vein, dilated congested portal tract (PT*), inflammatory cell infiltration, and apoptotic cells, which were most evident in the STZ high-dose group (60 mg/kg) (**B**, **C**). Stain: hematoxylin and eosin (H&E), magnification 200×. Abbreviations: STZ = streptozotocin; H&E = hematoxylin and eosin; PT = portal tract.

Liver tissues for all tested groups were illustrated in Fig. 7, the normal control group was showing normal looking central vein and portal tract formed of portal vein, hepatic artery and bile duct with normal sinusoids and healthy hepatocytes have central vesicular nuclei, others show prominent nucleolus (Fig. 7A). The other both groups who received the drug in two different doses were showing only dilated congested blood vessels (Fig. 7B and C).

#### Histopathological picture of the kidney tissue

**Fig. 8:**
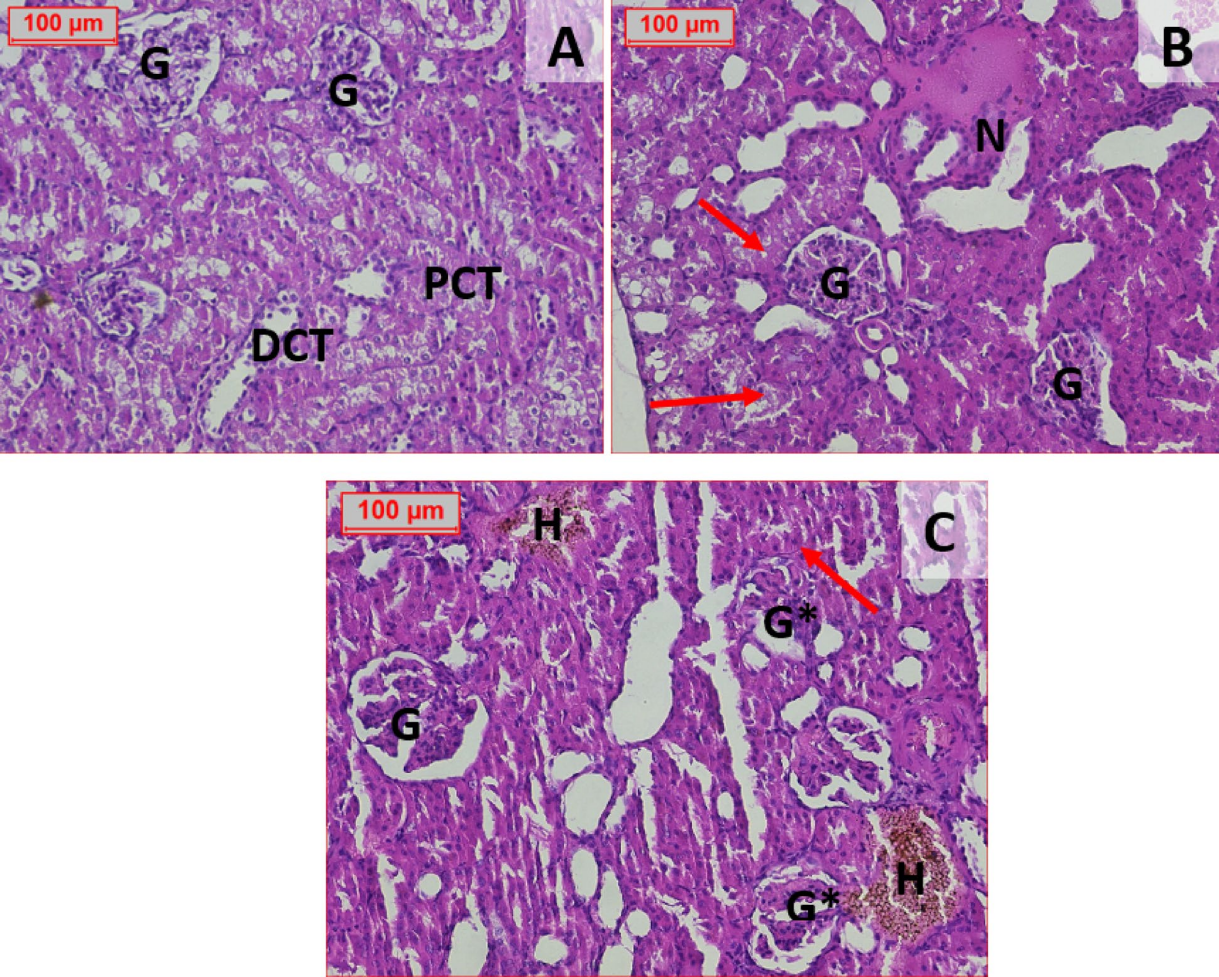
Effect of different administration of STZ on the histopathological picture of the kidney of male Sprague-Dawley rats. The examined kidney tissue in the normal control group showed a healthy appearance, with normal renal architecture formed of malpighian corpuscle which contain the glomerulus formed of capillary loops separated from Bowman capsule by Bowman Space, the proximal convoluted tubules lined by simple cuboidal with microvilli as it begins at the capsule and distal convoluted tubules (Fig. 8A). Sections from the second group (STZ 45 mg/kg) rats revealed some glomeruli are replaced by necrotic tissue with shedding of tubules lining showing intratubular debris (Fig. 8B). Shrinked glomerular tufts have been seen in the third group (STZ 60 mg/kg) also the proximal and distal tubules have disrupted their epithelial lining also we noticed foci of intertubular hemorrhage (Fig. 8C). (G): Normal Glomeruli, (G*): Glomeruli with shrinked tuft, (PCT): Proximal convoluted tubules, (DCT): Distal convoluted tubules, (Red arrows): intratubluar debris, (N): Necrosis, (H): Hemorrhage (H&E 200×)

The examined groups of kidney tissue as the normal control group showed a healthy appearance, with normal renal architecture formed of malpighian corpuscle which contain the glomerulus formed of capillary loops separated from Bowman capsule by Bowman Space, the proximal convoluted tubules lined by simple cuboidal with microvilli as it begins at the capsule and distal convoluted tubules (Fig. 8A). Sections from the second group (STZ 45 mg/kg) rats revealed some glomeruli are replaced by necrotic tissue with shedding of tubules lining showing intratubluar debris (Fig. 8B). Shrinked glomerular tufts have been seen in the third group (STZ 60 mg/kg) also the proximal and distal tubules have disrupted their epithelial lining also we noticed foci of intertubular hemorrhage (Fig. 8C).

#### Histopathological picture of the lung tissue

**Fig. 9:**
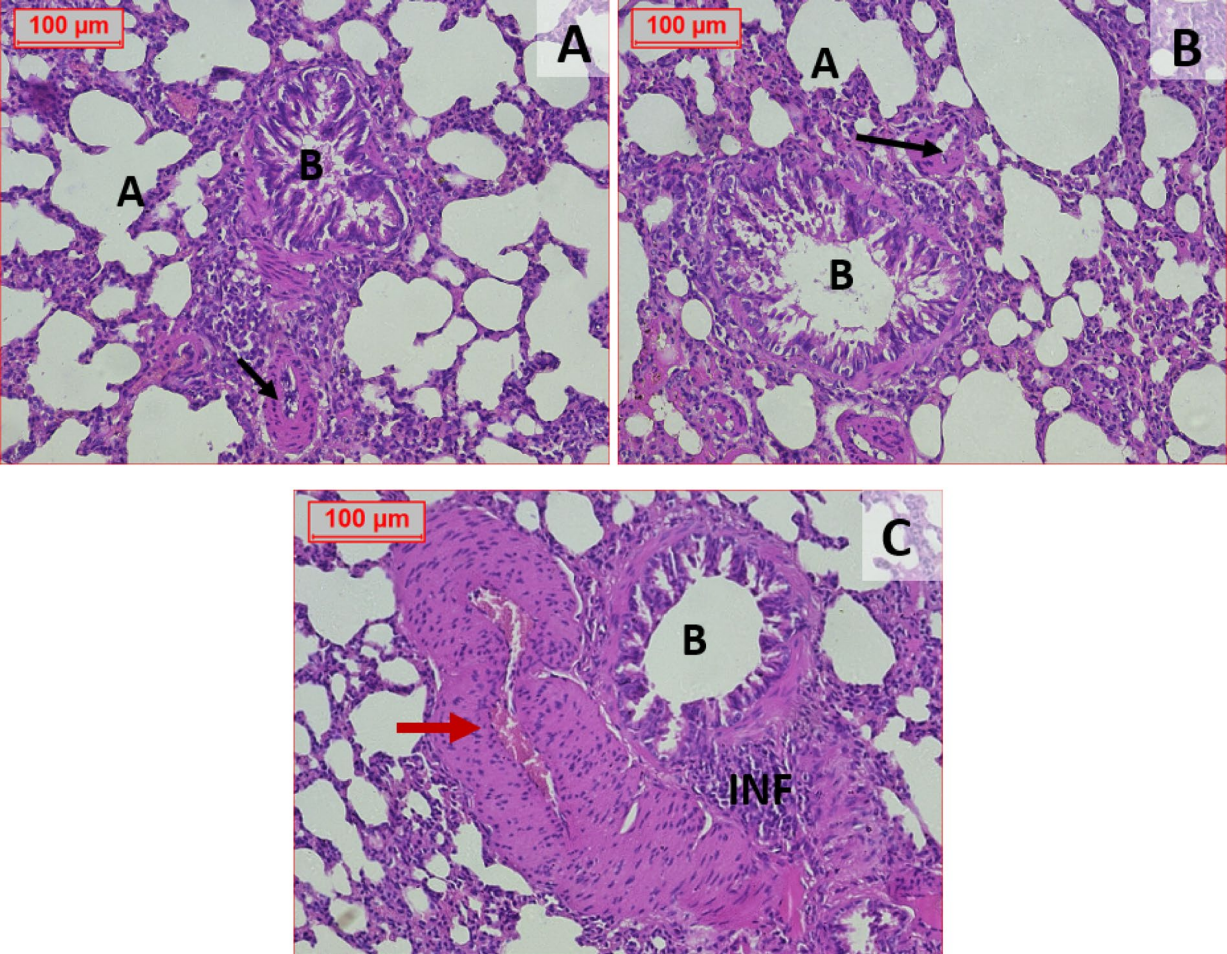
Effect of different administration of STZ on the histopathological picture of the lung of male Sprague-Dawley rats. Hematoxylin and eosin-stained sections of the lung tissue showing (normal control group) (Fig. 9A). and (STZ 45 mg/kg) (Fig. 9B) have normal looking architecture as the alveolar sacs separated by alveolar septa, comprising alveolar lining cells and thin-walled capillaries also the bronchiole has intact wall with normal looking ciliated columnar epithelium with normal looking bronchial vessels. On the contrary; the third group (STZ 60 mg/kg): noticed inflammatory reaction (↑↑↑INF; Inflammatory Foci) affecting vascular wall with marked hypertrophy and obliterated vascular lumina (Fig. 9C). (**A**): Normal Alveolar Sacs, (**B**): Bronchiole, (Red arrow): Normal Bronchial Vessels, (Black arrows): Hypertrophy and Obliterated Vessels, (INF): Inflammatory Foci (H&E 200×)

The examination of H&E stained sections of the lung tissue showing that the (normal control group) (Fig. 9A) and (STZ 45 mg/kg) (Fig. 9B) both were having normal looking architecture as the alveolar sacs will separated by alveolar septa, comprising alveolar lining cells and thin walled capillaries also the bronchiole has intact wall with normal looking ciliated columnar epithelium with normal looking bronchial vessels. The third group (STZ 60 mg/kg) just noticed inflammatory reaction affecting vascular wall with marked hypertrophy and obliterated vascular lumina (Fig. 9C).

#### Histopathological picture of the brain tissue

**Fig. 10:**
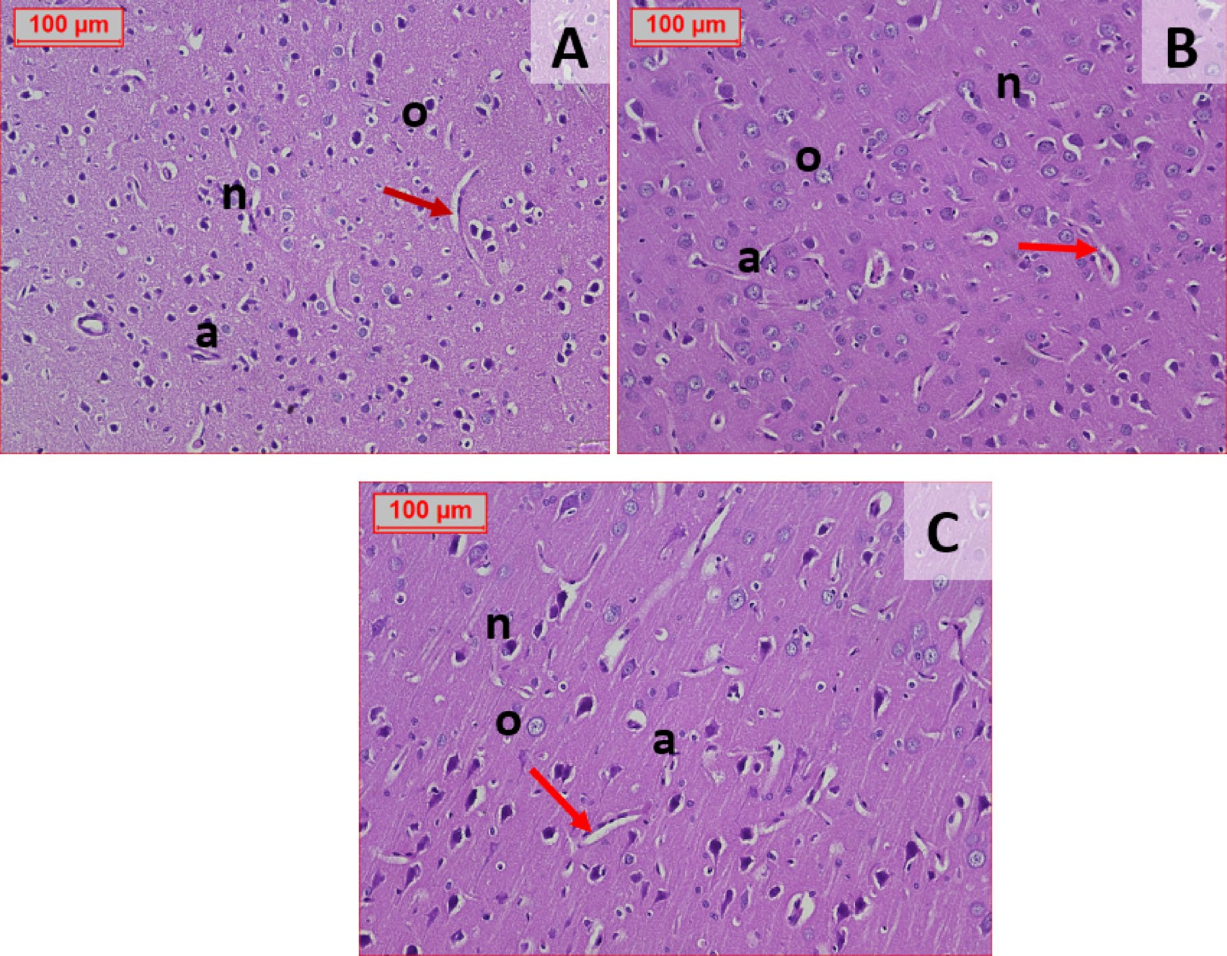
Effect of different administration of STZ on the histopathological picture of the brain of male Sprague- Dawley rats. Normal control group (Fig. 10A) showed normal histopathological picture of the cerebral cortex. STZ GROUP 45 showed also normal structure of cerebral cortex formed of neuronal cells; typically have large pale nuclei with prominent nucleoli. The non-neuronal cells (glial) cells include oligodendrocytes, hyperchromatic round nuclei with abundant clear cytoplasm (Fig. 10B). While STZ 60 Showed paler more elongated nuclei with scant cytoplasm, dilated and congested blood vessels (Fig. 10C). (n): Neuron Cells, (o): Oligodendrocytes, (**a**): Astrocytes, (Red Arrows): blood vessels. (H&E 200×)

The normal control group (Fig. 10A) exhibited a typical histopathological structure of the cerebral cortex. The STZ 45 group also showed a normal cortical architecture, with neuronal cells characterized by large, pale nuclei and prominent nucleoli. The non-neuronal (glial) cells, including oligodendrocytes, displayed hyperchromatic round nuclei with abundant clear cytoplasm (Fig. 10B). In contrast, the STZ 60 group demonstrated pathological changes, including paler, more elongated neuronal nuclei with scant cytoplasm, as well as dilated and congested blood vessels (Fig. 10C).

### Morphometric analysis

#### Effect of different administration of STZ on the nucleic acid content in liver, kidney, lung, and brain tissues in male Sprague-Dawley rats

**Fig. 11:**
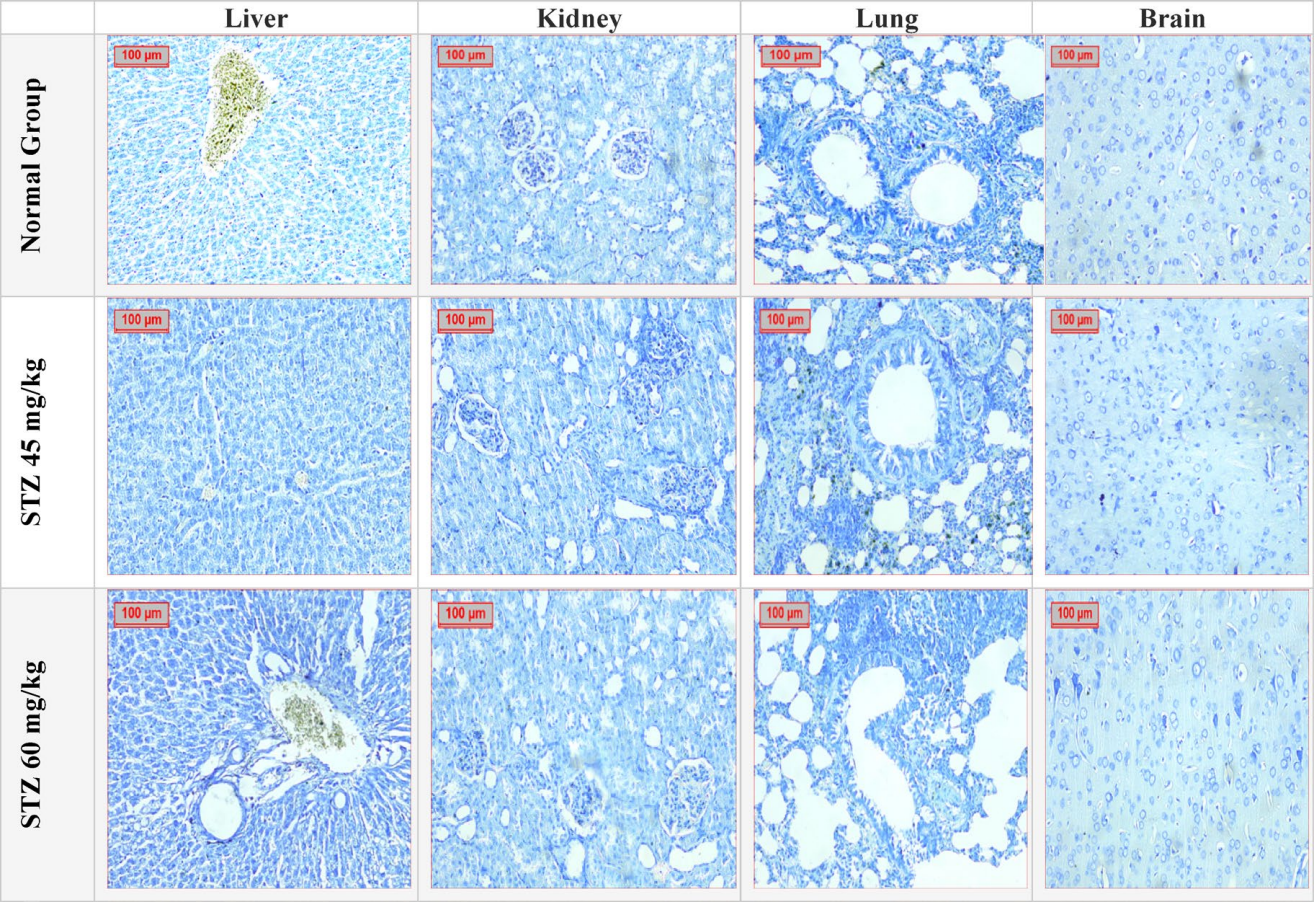
Effect of different administration of STZ on the nucleic acid content in liver, kidney, lung, and brain tissues in male Sprague-Dawley rats. A photomicrography of Liver, Kidney, Lung and Brain tissues for the tested groups; (**A**): Normal Control Group, (**B**): Group STZ 45, (**C**): Group STZ 60 (Toluidine blue stain 200×). Where, the nucleic acid content in liver, kidney, lung, and brain tissues were evaluated.

The study assessed the nucleic acid content in liver, kidney, lung, and brain tissues using Toluidine blue stain (Fig. 11)**,** by detection of area % of these contents with results expressed as mean ± SD in (Table 3)**.**

**Table 3:** Effect of different administration of STZ on the nucleic acid content in liver, kidney, lung and brain tissues.

	Liver	Kidney	Lung	Brain
Normal group	1.78 ± 0.17	7.60 ± 0.19	5.71 ± 0.29	2.19 ± 0.14
STZ 45 mg/kg	8.41 ± 0.72^a^	10.58 ± 0.28^a^	16.03 ± 0.5^a^	2.34 ± 0.36^a^
STZ 60 mg/kg	26.29 ± 0.51^b^	11.88 ± 1.13^b^	17.05 ± 1.3^b^	1.62 ± 0.07^b^

Results of Area % of nucleic acid content in liver, kidney, lung and brain tissues detected by Toluidine blue stain in all examined groups, Data were expressed as mean ± SD (8). Statistical analysis was carried out by one-way ANOVA. ^a^Significant difference from normal group *p* < *0.05*. ^b^Significant difference from STZ (45 mg/kg) group *p* < *0.05.*

The liver tissue of the normal group, contended 1.78 ± 0.17 of the nucleic acid. On the other hand, groups (STZ 45 & 60 mg/kg) showed significantly higher nucleic acid content in the liver 8.41 ± 0.72 and 26.29 ± 0.51, respectively.

According to Kidney tissue, the normal control has 7.60 ± 0.19 nucleic acid content, While Group 45 and Group 60 showed significantly higher nucleic acid content 10.58 ± 0.28 and 11.88 ± 1.13, respectively.

The normal control lung tissue has 5.71 ± 0.29, Groups (STZ 45 & 60 mg/kg) showed significantly higher nucleic acid content 16.03 ± 0.5 and 17.05 ± 1.3, respectively.

On the Other hand, there is no statistically difference of nucleic acids content between normal control group which has 2.19 ± 0.14 with the nucleic acids content of other two groups 2.34 ± 0.36 and 1.62 ± 0.07.

#### Effect of different administration of STZ on the connective tissue content in liver, kidney, and lung tissues in male Sprague-Dawley rats

**Fig. 12:**
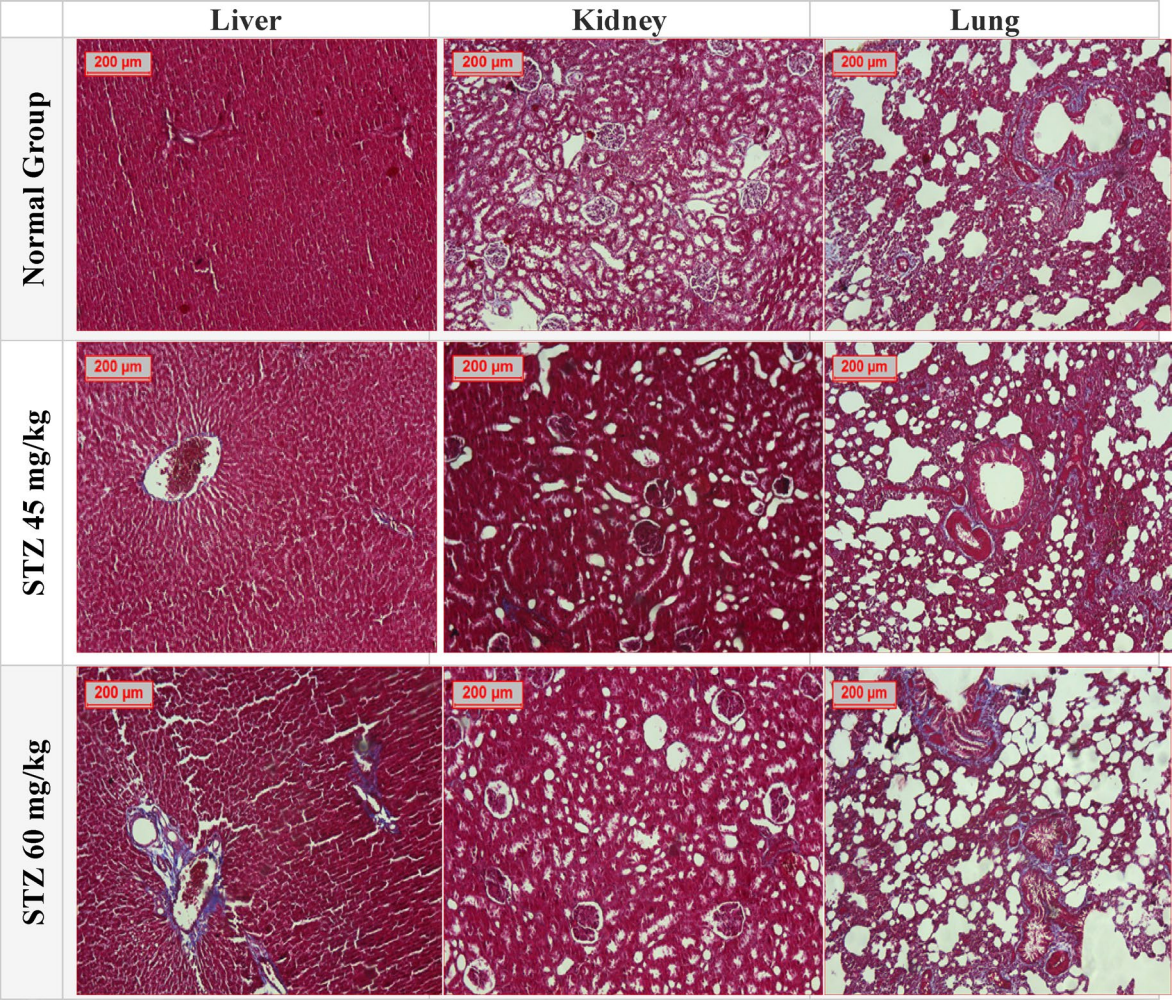
Effect of different administration of STZ on the connective tissue content in liver, kidney, lung, and brain tissues in male Sprague- Dawley rats. A photomicrography of Liver, Kidney and Lung tissues for the tested groups; (**A**): Normal Control Group, (**B**): Group STZ 45, (**C**): Group STZ 60 (Mallory trichrome stain 100×). Where, the connective tissue content in liver, kidney and lung tissues was evaluated.

The study measured the connective tissue content in liver, kidney, and lung tissues of different groups using Mallory trichrome stain (Fig. 12), by detection of area % of these contents and results expressed as mean ± SD in (Table 4)**.**

**Table 4:** Effect of different administration of STZ on the Area % of connective tissue content in liver, kidney, lung and brain tissues.

	Liver	Kidney	Lung
Normal group	6.82 ± 1.06	26.58 ± 0.67	36.47 ± 2.37
STZ 45 mg/kg	8.26 ± 0.89^a^	9.61 ± 0.57^a^	24.52 ± 0.83^a^
STZ 60 mg/kg	26..24 ± 5.3^b^	7.53 ± 0.84^b^	18.86 ± 0.94^b^

Results of Area % of connective tissue content in liver, kidney and lung tissues detected by Mallory trichrome stain in all examined groups, Data were expressed as mean ± SD (8). Statistical analysis was carried out by one-way ANOVA. ^a^Significant difference from normal group *p* < *0.05*. ^b^Significant difference from STZ (45 mg/kg) group *p* < *0.05.*

In the normal group, the liver had a connective tissue content of 6.82 ± 1.06, Group (STZ 45 mg/kg) showed connective tissue content of 8.26 ± 0.89 with no significant difference. Group (STZ 60 mg/kg) exhibited a significantly higher liver connective tissue content of 26.24 ± 5. According to the kidney tissue, the normal control had 26.58 ± 0.67 of connective tissue content which showed significantly lowering in other tested groups, as connective tissue content of groups (STZ 45 and 60 mg/kg) were 9.61 ± 0.57, and 7.53 ± 0.84, respectively. The normal control connective tissue in lung had 36.47 ± 2.37. A significantly lower lung connective tissue content of both treated groups by different concentration of 24.52 ± 0.83 & 18.86 ± 0.94, respectively.

## Discussion

Diabetes mellitus (DM) is a chronic metabolic disorder with rapidly increasing global prevalence. Nearly half a billion individuals worldwide were reported to be diabetic, with projections estimating a 25% increase by 2030 and 51% by 2045^29^. Experimental models using streptozotocin (STZ), a naturally occurring nitrosourea compound produced by Streptomyces achromogenes, remain widely employed to investigate DM pathophysiology due to STZ’s selective cytotoxicity toward pancreatic β-cells^9,30^. STZ acts via DNA alkylation and generation of reactive oxygen species (ROS), leading to β-cell necrosis and subsequent insulin deficiency. Notably, higher doses of STZ (60 mg/kg) induce more profound β-cell destruction compared to lower doses (45 mg/kg), resulting in more severe metabolic and inflammatory disturbances^12,31^. STZ elicits an immune and inflammatory reaction, presumably concerned to the release of glutamic acid decarboxylase autoantigens^30^. Remarkably, STZ has a short half-life, it is metabolized rapidly in the liver and eliminated by renal excretion. The consequences of diabetic hyperglycemia may be linked to additional functional impairment of the kidney or liver after STZ has been removed from the body^32^. This serves as the foundation for research on the mechanisms of STZ diabetes complications in liver and kidney as well as other organs such brain, and lung.

In the current study, a single intraperitoneal administration of STZ at two distinct doses (45 and 60 mg/kg) induced characteristic diabetic phenotypes in rats. Both doses resulted in significant impairment of β-cell function, as evidenced by elevated blood glucose and glycated hemoglobin (HbA1c), reduced insulin levels, and weight loss. However, the 60 mg/kg group exhibited more pronounced changes across these metabolic parameters, consistent with dose-dependent cytotoxicity. STZ enters β-cells through GLUT2 transporters and causes extensive DNA damage and NAD⁺ depletion, with the extent of β-cell loss directly proportional to the administered dose^10^. Furthermore, the higher dose likely overwhelmed compensatory repair mechanisms, exacerbating insulinopenia and hyperglycemia. This aligns with pervious findings that STZ-induced diabetes in rats leads to increased blood sugar which due to oxidative stress and pancreatic β cell destruction consequently, attributed to insulin deficiency and activates glucose-6-phosphatase, an enzyme crucial for gluconeogenesis, thus increasing blood sugar levels^33,34^.

Lipid profile disturbances were also more severe in the STZ 60 mg/kg group, with significantly elevated triglycerides (TG), total cholesterol (TC), and low-density lipoprotein (LDL-C), alongside increased high-density lipoprotein (HDL-C). These findings align with prior studies showing that insulin deficiency enhances hepatic lipogenesis, promotes mobilization of free fatty acids from adipose tissue, and reduces lipoprotein lipase activity, leading to diabetic dyslipidemia. These observations were consistent with earlier research confirmed that dyslipidemia associated with diabetes. This dyslipidemia occurs due to the release of fatty acids from adipose tissue and the liver as a result of insulin deficiency, which impairs glucose utilization. The surplus fatty acids are then transformed into phospholipids and cholesterol in the liver, exacerbating hyperlipidemia. Furthermore, hyperglycemia in diabetic rats produces reactive oxygen species that can cause lipid peroxidation and damage to cell membranes^35^.

Concomitantly, chronic hyperglycemia is considered the most contributor to the generation of intracellular reactive oxygen species (ROS), leading to diverse diabetic complications. Besides, the complications are related with the inability of cells to maintain glucose homeostasis herein cause an increase in glycolysis and ROS production resulting in oxidative lesion, protein denaturation, lipid peroxidation, and damage to mitochondrial DNA which induces cell dysfunction^36^. In line with the data of previous work, these events were confirmed with a significant depletion of antioxidant GSH and elevation of MDA which is one of the final products of polyunsaturated fatty acids peroxidation in diabetic rats particularly in the 60 mg/kg group^37^.

The results of the present study indicated that intraperitoneal injection of STZ activates the cascade of inflammatory signaling pathways that characterized by raised in serum levels of inflammatory cytokines TNF-α and il-6, particularly in the higher dose group. These results are in accordance with former research which stated that oxidative stress induces the overproduction of ROS, and activates the inflammatory cascades herein contribute to encouraging the inflammation consequences. STZ-induced hyperglycemia promotes inflammatory signaling via NF-κB activation, triggering cytokine release and immune cell infiltration. The observed proinflammatory state is consistent with previous reports demonstrating that oxidative stress and inflammation synergistically drive diabetes-related tissue injury^38^.

In addition to metabolic disruption, STZ toxicity extended to hepatic and renal tissues, as indicated by elevated serum AST and ALT levels. The higher dose group again exhibited more severe hepatic enzyme elevations, likely due to aggravated oxidative and inflammatory damage. These findings support prior reports linking diabetic hyperglycemia to hepatocellular injury and altered liver enzyme profiles^39,40^.

Furthermore, the histopathological examination confirmed multi-organ damage in STZ-treated rats, including the liver, kidneys, lungs, and brain, with more severe alterations observed in the STZ 60 mg/kg group. Recent studies have also demonstrated STZ-induced damage across multiple organs, reflecting systemic toxicity and sustained hyperglycemia^40–44^.

An important yet previously underexplored observation in this study was the significant increase in nucleic acid content in liver and lung tissues, in contrast to the absence of significant change in brain tissue. This finding highlights distinct tissue-specific responses to STZ toxicity, which may reflect differential sensitivity to DNA damage, cellular turnover rates, or compensatory transcriptional activity. The liver and lung are highly vascularized and metabolically active organs, making them more susceptible to systemic toxicants such as STZ. The observed increase in nucleic acid levels may result from enhanced transcriptional activity or a regenerative response to cellular injury, including increased proliferation of parenchymal or immune cells. These tissues also possess higher baseline capacities for nucleic acid synthesis, which may be upregulated in response to oxidative or nitrosative stress induced by STZ. In contrast, brain tissue did not exhibit significant changes in nucleic acid content. This may be attributed to the relative impermeability of the blood–brain barrier, which limits STZ uptake into the central nervous system. Additionally, the brain has a lower proliferative index compared to peripheral organs, resulting in a more stable nucleic acid profile even under systemic stress. These organ-specific differences in nucleic acid content following STZ administration suggest that tissue vulnerability is modulated not only by STZ biodistribution but also by intrinsic regenerative and metabolic characteristics. These observations were along with the histopathological results of earlier studies which emphasize that diabetes leads to significant histological changes in aforementioned organs in diabetic animal models^40,44,45^.

## Conclusion

In summary, our data confirm that STZ-induced diabetes manifests in a dose-dependent manner, with 60 mg/kg producing more severe metabolic, oxidative, and histological abnormalities than 45 mg/kg. These results emphasize the importance of STZ dose selection in experimental diabetes models and provide new insight into the tissue-specific effects of STZ toxicity.

## Data Availability

All data are available upon request from the corresponding author.
